# Microbial Colonization of Pneumatic Tourniquets in the Orthopedic Operating Room

**DOI:** 10.7759/cureus.5308

**Published:** 2019-08-02

**Authors:** Syed H Mufarrih, Nada Q Qureshi, Rizwan H Rashid, Bilal Ahmed, Seema Irfan, Akbar J Zubairi, Shahryar Noordin

**Affiliations:** 1 Orthopedic Surgery, Aga Khan University, Karachi, PAK; 2 Cardiology, Aga Khan University, Karachi, PAK; 3 Medicine, Aga Khan University, Karachi, PAK; 4 Microbiology, Aga Khan University, Karachi, PAK

**Keywords:** tourniquet, surgical site infection, joint arthroplasty, operating room, infection

## Abstract

Background

The rate of surgical site infections following orthopedic procedures is approximately 2% globally. Potential sources of contamination in the operating room include pneumatic tourniquets, blood pressure cuffs, and stethoscopes, among others. Our study aims to investigate microbial colonization on reusable pneumatic tourniquets stored and used in the orthopedic department of our institution and evaluate the efficacy of the cleaning protocols employed.

Methods

Over a course of two weeks, 26 samples were obtained. A total of 14 pneumatic tourniquets were sampled preoperatively on Monday morning following the weekly cleaning protocol of soaking the tourniquets in sodium hypochlorite for 30 minutes while 12 tourniquets were cultured immediately following the postoperative cleaning protocol of wiping the tourniquet clean with a cloth soaked in sodium hypochlorite. Samples were cultured on MacConkey and sheep blood agar and incubated at 37-degrees centigrade for a total of 48 hours. Organisms were identified and colony count was documented. The analysis was performed using the Fisher Exact test on SPSS v23 (IBM Corp., Armonk, NY, US).

Results

All 14 samples obtained after being soaked in sodium hypochlorite for 30 minutes cultured negative. However, four out of 12 (33%) samples obtained after simply wiping the pneumatic tourniquet with a cloth soaked in sodium hypochlorite cultured coagulase-negative Staphylococci. The difference between the two was significant (p=0.002).

Conclusion

Postoperative tourniquets, wiped with a cloth soaked in sodium hypochlorite and ready to be used on the next patient, were found to be contaminated with coagulase-negative Staphylococcus. This species is notorious for causing surgical site infections following implant-related surgeries potentially through direct inoculation and cross-infections intraoperatively and in storage. Efforts to identify the relationship with postoperative surgical site infections need to be made to suggest more aggressive cleaning protocols.

## Introduction

Surgical site infections (SSIs) are an unfortunate complication of extremity surgery, even more so where implants are involved such as in fracture fixation and joint arthroplasty. Besides the significant morbidity associated with surgical site infections, the economic implications are huge owing to the increase in the length of hospital stay, need for revision surgery, and multiple hospitalizations, collectively contributing to an approximately 300% increase in the cost of surgery [1]. Given the current SSI rate of approximately 2% [2-3] following orthopedic procedures and the potentially preventable nature of the complication, it is imperative to direct all efforts to tackle this issue head-on.

Previous literature has identified stethoscopes [4-6], mobile phones [5], marking pens [7], and blood-pressure cuffs [7] as possible sources of infection in the operating room.

The reusable pneumatic tourniquet, a “non-critical” item as per the Spaulding classification, has been highlighted as a possible source of infection following orthopedic procedures [8]. Pneumatic tourniquets are employed in orthopedic procedures to maintain a bloodless field for the duration of the surgery. Previous studies have shown that many tertiary care hospitals lack a standard protocol for the cleaning of the tourniquets [9], and the same tourniquet may be used on patients with both clean and contaminated wounds. Subsequently, contaminated tourniquets may be stored with the other tourniquets without adequate disinfection. Hence, we hypothesized that pneumatic tourniquets maybe serve as a potential reservoir for pathogenic bacteria.

The aim of our study was to investigate whether reusable pneumatic tourniquets are colonized by microbes implicated in postoperative surgical site infections following orthopedic procedures and evaluate the efficacy of the pre and postoperative cleaning protocols employed at our institution.

## Materials and methods

Study design

A cross-sectional study was conducted at the orthopedic operating rooms of Aga Khan University Hospital (AKUH), Karachi, from August 2017 to September 2017. Preoperative and postoperative samples were collected from the 14 pneumatic tourniquets stored in the orthopedic operating rooms' storage unit. The purpose of this study was to investigate the microbial colonization on pneumatic tourniquets employed in extremity surgery.

Sampling

Materials

At our university hospital, 14 reusable pneumatic tourniquets are stored together in a pneumatic box for use in the orthopedic department exclusively. The available tourniquets had a range of sizes, with an outer fabric layer with velcro and an inner silicone bladder. An inflatable balloon made of latex covered the entire length. These tourniquets are used in most orthopedic surgeries in close proximity to the surgical site throughout the length of the procedure.

Timings of Pre and Postoperative Sampling

Two types of cleaning protocols are employed at our institution. Once weekly, all tourniquets are soaked in sodium hypochlorite for 30 minutes. Between surgeries, after being used on one patient before moving on to the next, the tourniquet is simply wiped with a cloth soaked in sodium chlorite. We obtained “preoperative” samples following the once-weekly cleaning protocol on Monday morning and “postoperative” samples following the wiping of the tourniquet with sodium hypochlorite. This was done to assess the adequacy of each protocol. Tourniquets employed in surgical debridement were not used in our study, as they are classified as “dirty surgeries.” During the course of sampling, two tourniquets at the extremes of sizes were not employed in any procedure and, therefore, their postoperative sample was not obtained.

Procedure

**Figure 1:**
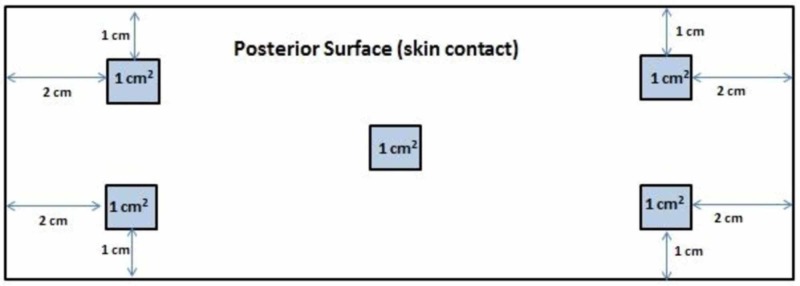
Technique for obtaining sample swabs from pneumatic tourniquets.

In collaboration with the microbiology department of our institute, a standardized protocol for sample collection from each tourniquet was developed. An orthopedic resident, trained in the sample collection technique by the microbiology department was responsible for obtaining all samples. Necessary measures to prevent cross-infection, such as the use of gloves and a mask, were taken during sampling. A single swab on a stick soaked in normal saline was used to wipe a one-centimeter square area from five points on the posterior surface (which has contact with the patient skin) of each tourniquet. Four points were each at a distance of one centimeter from the four edges, with the fifth one at the center (Figure 1). The swab was then sealed in a test tube containing normal saline and was sent for culture to the microbiology department and processed within 24 hours without any storage phase.

Laboratory procedure

The sample swabs were cultured on both MacConkey and sheep blood agar and incubated at 37-degree centigrade for 24 hours. If no growth was seen, the culture plate was reincubated for an additional 24 hours. In cases of bacterial growth, the colony-forming units (CFUs) on the culture plate were counted. Finally, culture mediums were tested for the growth of Staphylococcus species, including Staphylococcus aureus (MSSA/MRSA) and coagulase-negative Staphylococcus (CoNS), Pseudomonas species, Acinetobacter species, Streptococcus species, and Klebsiella species. Growth on the culture media was documented as the bacterial species with colony-forming units.

Statistical analysis

The results of preoperative and postoperative cultures from all tourniquets have been tabulated. Data were analyzed using SPSS version 22 (IBM Corp., Armonk, NY, US). Simple frequencies and proportions were calculated to describe categorical data. To calculate the 95% confidence intervals (CIs) for the proportion of tourniquets getting contaminated postoperatively, the Fisher Exact test was used.

## Results

All 14 preoperative samples did not culture any microorganisms. Out of the 12 postoperative samples, four (33.3%) were contaminated. Those that were contaminated had a colony count between one and two colony-forming units. Coagulase-negative Staphylococcus was the only organism isolated (Table 1). The difference between preoperative and postoperative contamination was significant. The postoperative decontamination protocol of wiping the tourniquet with a cloth soaked in sodium hypochlorite is not effective when compared to the preoperative decontamination protocol of soaking the tourniquet in the sodium hypochlorite solution (p=0.002).

**Table 1:** Qualitative and quantitative results of growth on culture media.

Tourniquet	Pre-operative	Post-operative	Colony Forming Units (CFUs)
1	Not isolated	Not Isolated	
2	Not isolated	Sample not sent	-
3	Not isolated	Not isolated	-
4	Not isolated	Coagulase-negative Staphylococcus	1
5	Not isolated	Not isolated	-
6	Not isolated	Not isolated	-
7	Not isolated	Coagulase-negative Staphylococcus	1
8	Not isolated	Sample not sent	-
9	Not isolated	Not isolated	-
10	Not isolated	Coagulase-negative Staphylococcus	2
11	Not isolated	Not isolated	-
12	Not isolated	Coagulase-negative Staphylococcus isolated	2
13	Not isolated	Not isolated	-
14	Not isolated	Not isolated	-

## Discussion

With the reported increase in life expectancy by the World Health Organization (WHO), osteoarthritis is expected to become the fourth leading cause of disability by 2020 [10]. Needless to say, the incidence of arthroplasty, which is the gold standard for the treatment of end-stage osteoarthritis, is expected to rise significantly [2,11]. According to available literature, the incidence of postoperative surgical site infections for joint arthroplasty is approximately 2% [12-15]. These statistics are alarming when the devastating consequences of an SSI, including increased length of hospital stay, with more than double the hospital costs and higher complication and mortality rates [13], are taken into account.

Despite the advancement of medical science, the war against infection continues to persist. Pneumatic tourniquets, classified as “non-critical” items by the Spaulding classification, have been implicated as a source of contamination in the operating room [16]. Changing the resistance patterns of microbes and accountability for the biofilm activity of the organisms may push for items, including tourniquets, stethoscopes, and marking pens, among others, to demand more aggressive cleaning protocols [17-19].

The use of pneumatic tourniquets in joint arthroplasty is now predominantly part of the standard procedure, as it benefits surgeons by maintaining a bloodless field, improving the visualization of important structures, strengthening the bone cement interface, and expediting the procedure [20]. However, recent studies have shown that reusable tourniquets are indeed contaminated and have postulated their potential role in the development of surgical site infections [8,21-22].

Compared to previous studies, which have reported contamination of up to 80% of the tested tourniquets with much higher colony counts, contamination in our study is low [8,23-24]. An obvious reason is that our study intended to investigate the efficacy of cleaning protocols and thus obtained samples after some cleaning mechanism had been employed. Other possible contributors for this difference may be the exclusion of cases of surgical debridement in our study and the inclusion of trauma-related orthopedic procedures in the referenced study.

Coagulase-negative Staphylococcus was the only organism isolated from our tourniquets. Although a part of the normal flora of the skin and mucous membranes, CoNS is among the most common causes of surgical site infections in orthopedic surgery along with Staphylococcus aureus, Enterococci, and Streptococci [24-27]. They are now gaining importance as nosocomial pathogens due to the emergence of methicillin-resistant strains, which may act as reservoirs for genetic material, particularly SCCmec IVa, found in MRSA [28-29].

Isolating this notorious species from tourniquets that were ready to be used in the next procedure is alarming, as the contaminated tourniquets may be a source of surgical site infections through various mechanisms. Firstly, direct inoculation of the surgical site by the bacteria on the tourniquet can occur through staff who fail to follow a proper hand hygiene protocol due to the non-critical status of the pneumatic tourniquet [30]. The use of a contaminated tourniquet can go on to serve as a source of infection in all subsequent surgeries until the standard weekend decontamination is performed. This phenomenon of cross-infection has been reported in previous studies as well [8,24]. Furthermore, the same tourniquet when stored in a pneumatic box with the rest may contaminate the other tourniquets, particularly if the scenario involves use in a traumatic or wound debridement surgery.

To decrease the rate of surgical site infections, efforts should be made to establish the relationship of colonization to surgical site infections, explore the contamination of other items in the operating room, such as marking pens, blood pressure cuffs, and stethoscopes, and investigate the efficacy of cost-effective cleaning methods.

The findings of our study are based on the cultures taken from a single center. To draw definitive conclusions, the study needs to be replicated in other centers. Multiple batches may be tested if the number of tourniquets available is small. Furthermore, in order to establish a causal relationship between colonization and infection, methods for the typing of bacterial strains, such as pulse-field gel electrophoresis, need to be utilized.

## Conclusions

Re-usable pneumatic tourniquets cleaned by wiping with a cloth soaked in sodium hypochlorite may still be contaminated with potentially pathogenic bacteria when compared to soaking the tourniquet in sodium hypochlorite for 30 minutes. The colonization of pneumatic tourniquets may contribute to surgical site infections following orthopedic procedures in several ways, including direct inoculation, breaching of hand hygiene protocol, and cross-infection. Efforts to establish a causal relationship between contamination and surgical site infections need to be made before suggesting more aggressive cleaning protocols.
